# Beyond da Vinci: a systematic review of next-generation multiport robotic platforms in pediatric surgery

**DOI:** 10.1007/s11701-026-03879-4

**Published:** 2026-09-07

**Authors:** Donatella Di Fabrizio, Giovanni Cobellis, Edoardo Bindi

**Affiliations:** 1 Department of Specialized Clinical and Odontostomatological Sciences, University Politecnica of Marche, Ancona, Italy; 2 Pediatric Surgery Unit, Salesi Children’s Hospital, Ancona, Italy

**Keywords:** Pediatric surgery, Robot-assisted surgery, Robotic platforms, Senhance, Hugo, Hinotori

## Abstract

As robotic surgery expands beyond the da Vinci platform, the relevance of new-generation systems to pediatric patients remains uncertain. This review examined the technical characteristics, applications, and perioperative outcomes of alternative multiport robotic platforms in children. PubMed/MEDLINE, Web of Science, Scopus, and the Cochrane Library were searched through 28 February 2026 in accordance with PRISMA 2020. Owing to clinical heterogeneity, findings were synthesized narratively, with Wilson 95% confidence intervals for key binary outcomes. Six studies reported 166 patients across 27 procedure types. Senhance accounted for 164 patients, while Hugo RAS and Hinotori were each represented by one patient. Ages ranged from 15 days to 17 years and weights from 3.8 to more than 100 kg. Senhance was the only platform used with 3-mm robotic instruments. Conversion or planned escalation occurred in 19 patients (11.4%; 95% CI, 7.5–17.2%). Four intraoperative complications were reported (2.4%; 95% CI, 0.9–6.0%). Across all reports, 20 patients experienced postoperative complications (12.0%; 95% CI, 7.9–17.9%). In the largest cohort, seven patients required reintervention (4.6%; 95% CI, 2.2–9.2%) and seven were readmitted (4.6%; 95% CI, 2.2–9.2%). No deaths or comparative pediatric studies were reported. Published experience remains sparse and is almost entirely limited to Senhance. Current evidence describes early clinical use without establishing comparative safety, effectiveness, or platform superiority. Prospective multicenter studies with standardized reporting are needed.

**Supplementary Information:**

The online version contains supplementary material available at https://doi.org/10.1007/s11701-026-03879-4.

## Introduction

Robotic-assisted surgery has transformed adult surgical practice over the past two decades, yet its integration into pediatric care has followed a markedly slower and more constrained trajectory [1, 2]. The Da Vinci Surgical System (Intuitive Surgical) dominates the published pediatric literature, with pyeloplasty, Nissen fundoplication, and cholecystectomy accounting for the majority of reported cases [3–5], but the platform carries a structural limitation that has consistently restricted its reach across the pediatric age spectrum: its smallest available instruments measure 8 mm in diameter, and its endoscopes are 8 or 12 mm [6]. In a child weighing less than 5 kg, these dimensions often render trocar placement geometrically limited, the intra-abdominal working space becomes critically restricted, and the rigidity of a single docked four-arm cart conflicts with the spatial reality of a small thoracic or abdominal cavity [7].

The surgical robotics market has diversified substantially over the past decade. Several new-generation multiport platforms have received regulatory clearance, such as the Senhance Surgical System (SSS; Asensus Surgical), Hugo RAS (Medtronic), the Hinotori Surgical Robot System (Medicaroid), Versius (CMR Surgical), and Revo-i (Meerecompany), each one incorporating distinct engineering choices around modularity, console design, instrument reusability, and instrument diameter [8, 9]. For example, the SSS, with its 3 mm instrument portfolio, was engineered with small-cavity surgery explicitly in view [10]; the Hugo RAS, with its four-cart modular architecture and freely adjustable docking angles, prioritises flexibility for complex multi-quadrant procedures [11]; the Hinotori prioritises Japanese surgical workflow integration and compact articulated arm design [12]. Whether and how these features translate into pediatric clinical practice has never been systematically assessed.

To date, no structured synthesis has specifically examined the pediatric experience with these systems from a technical perspective. A systematic review is therefore needed to define the current state of the field several years after the introduction of these new platforms. The aim of this study was to systematically review the available pediatric literature on new-generation multiport robotic platforms, with particular focus on their technical characteristics, reported surgical applications, perioperative outcomes, and the main technical strengths and limitations emerging from early clinical experience.

## Methods

### Protocol and registration

This systematic review was conducted and reported in accordance with the Preferred Reporting Items for Systematic Reviews and Meta-Analyses (PRISMA 2020) statement [13]. The protocol was prospectively registered on PROSPERO prior to study selection, and no amendments to the registered protocol were made (CRD420261308934).

### Eligibility criteria

Eligibility criteria were defined a priori according to the population, intervention, comparator, outcomes (PICO), and study design framework. Suitable studies enrolled patients aged 0–18 years undergoing minimally invasive robotic-assisted surgery with any new-generation multiport robotic platform other than the Da Vinci Surgical System, including the SSS, Hugo RAS, Hinotori, Versius, Revo-i, KangDuo, Toumai, Avatera, and Dexter. Studies reporting mixed adult and pediatric cohorts were eligible when individual pediatric patient data were separately extractable. No comparator was required for inclusion. Eligible study designs included randomized controlled trials, prospective and retrospective cohort studies, case series, and case reports with at least one extractable perioperative, postoperative, or technical feasibility outcome. Reviews, editorials, letters, conference abstracts without full-text data, and preclinical, cadaveric, or animal studies were excluded. No restriction on country or clinical setting was applied. The English-language restriction was prespecified to permit consistent full-text assessment and data extraction across reviewers; its potential to introduce language bias was considered in the interpretation of the findings.

### Search strategy

A comprehensive search was conducted in MEDLINE via PubMed, Web of Science, Scopus, and the Cochrane Library through 28 February 2026. The search combined platform-specific identifiers with pediatric population terms. The core search structure included terms related to pediatric populations, such as pediatric, paediatric, child, infant, neonate, and adolescent, combined with the names of the robotic systems of interest, including Senhance, Hugo RAS, Versius, Revo-i, Hinotori, KangDuo, Toumai, Avatera, and Dexter. Reference lists of included articles were also screened manually to identify any additional eligible studies.

### Study selection and data extraction

Two reviewers independently screened all titles and abstracts against the eligibility criteria, followed by independent full-text assessment of potentially eligible records. After removal of duplicates, titles and abstracts were screened against the predefined eligibility criteria. Full texts of potentially relevant studies were then assessed for final inclusion. Reasons for exclusion at full-text stage were recorded. Particular attention was paid to possible overlap of study populations, assessed by comparing institutions, recruitment dates, procedure mix, sample size, robotic platform, and author lists. When multiple reports described the same or partially overlapping cohorts, these publications were collated and treated as a single study unit. In such cases, the most comprehensive and methodologically informative report was retained as the primary source for synthesis. This approach was adopted to avoid double counting of patients and to preserve the integrity of the synthesis. Data extraction was performed using a standardized form specifically designed for this review. Extracted variables included study characteristics, such as author, year of publication, country, study design; patient characteristics, including sample size, age, sex, weight, and relevant comorbidities, the robotic platform used and procedure performed. Perioperative outcomes included surgeon training or proctorship, trocar size and number, need for accessory ports, operative time, docking time when available, blood loss, conversion to laparoscopy or open surgery, intraoperative and postoperative complications, length of hospital stay, and short follow-up outcomes when reported.

### Risk of bias assessment

Given the anticipated predominance of case series and observational cohorts, methodological quality was assessed using design-appropriate tools: the Joanna Briggs Institute critical appraisal checklist for case series and case reports [14], and the ROBINS-I tool for non-randomized comparative studies [15]. No randomized controlled trials were identified.

### Data synthesis

Clinical and methodological heterogeneity across platforms, procedures, and age groups precluded formal meta-analysis. A structured narrative synthesis was performed, organized by robotic platform and procedural category. Continuous outcomes are reported as means with standard deviations or ranges as provided in the source publications. For key binary outcomes, proportions and 95% confidence intervals were calculated using the Wilson method. These intervals quantify precision only and were not used for cross-platform inference. All platform-level findings were treated as descriptive, and no direct or indirect cross-platform comparison was undertaken. Formal assessment of publication bias was not performed because only six heterogeneous descriptive studies were included and no common comparative effect estimate was available.

## Results

### Study selection

**Fig. 1:**
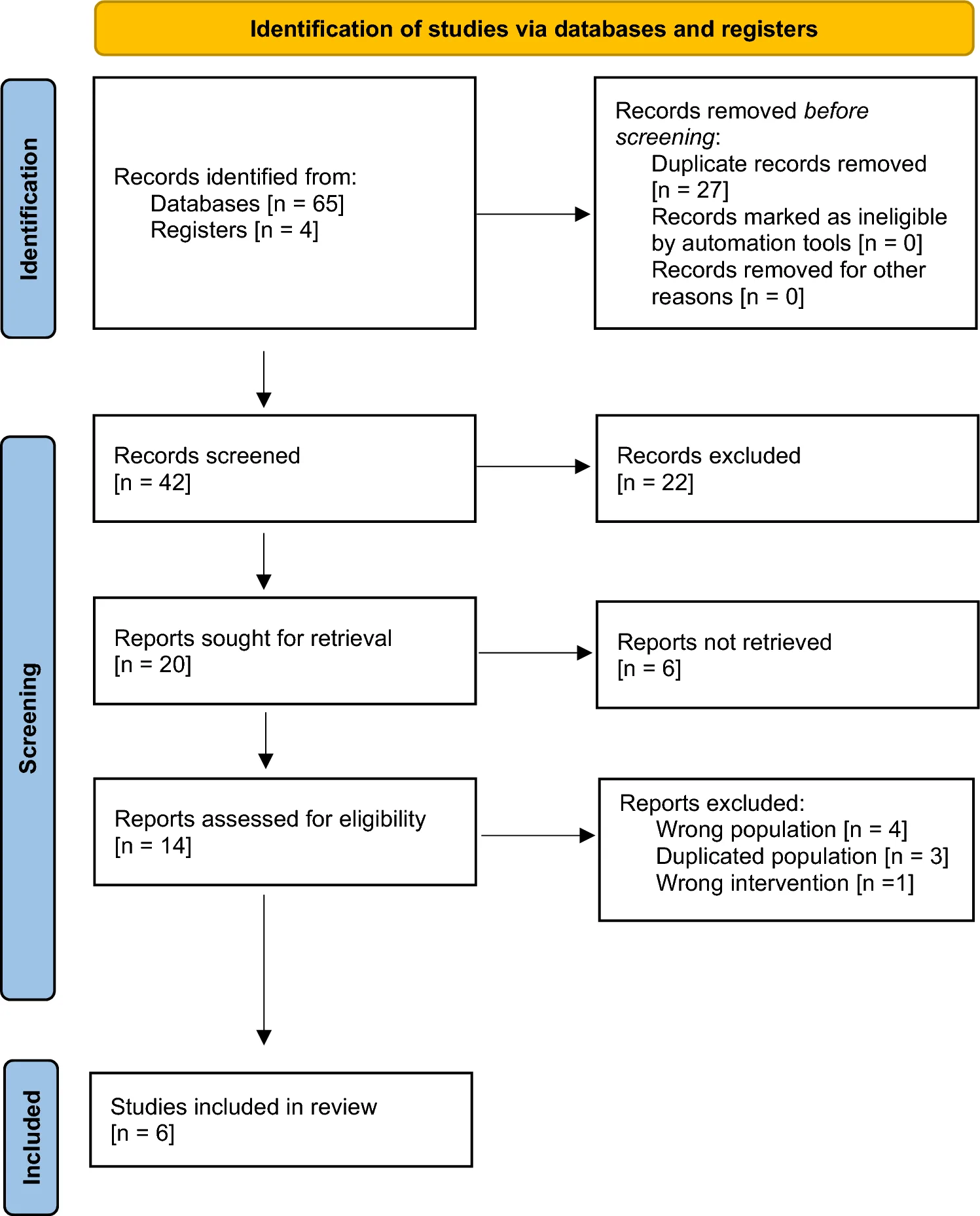
Study selection process according to the PRISMA 2020 flow diagram

The database search identified 69 records, including 65 records from bibliographic databases and 4 records from the Cochrane Library register. After removal of 27 duplicates, 42 records underwent title and abstract screening. Twenty full-text records were sought for assessment, and 14 were assessed for eligibility. Eight records were excluded: 4 because they included adult-only populations without extractable pediatric data, 3 because they reported duplicated populations already captured in a larger included cohort, and 1 because of wrong intervention. Six studies met the inclusion criteria and were included in the final synthesis (Fig. 1) [16–21].

### Characteristics of the included evidence

The 6 included studies were published between 2022 and 2026 and reported 166 pediatric patients who underwent surgery with new-generation multiport robotic platforms. The evidence base consisted of 2 case reports [16, 20], 3 case series [17–19], and 1 retrospective multicenter cohort [21], contributing 152 of 166 patients overall (91.6%) and 152 of 164 Senhance cases (92.7%); aggregate estimates therefore largely reflect this single study. Five studies were single-center reports [16–20], and 1 was a multicenter study [21]. No comparative pediatric study was identified. Analyzing the risk of bias, three studies were rated as low risk of bias [16, 18, 20] and three as moderate risk [17, 19, 21]; no study reached serious or critical risk. Detailed item-level results are reported in supplementary materials Table 1.

**Table 1:** Distribution of pediatric procedures performed with new-generation multiport robotic platforms

Procedure	Total [n]	Platform
**Abdominal Wall**	**48**	
○ Inguinal hernia repair	48	Senhance
**Upper gastrointestinal, hepatobiliary and splenic**	**72**	
○ Nissen fundoplication	40	Senhance
○ Cholecystectomy	20	Senhance
○ Splenectomy / resection of splenic cyst	5	Senhance
○ Heller-Dor procedure	3	Senhance
○ Resection of esophageal duplication cyst	2	Senhance
○ Esophagectomy	1	Senhance
○ Resection of choledochal cyst	1	Senhance
**Colorectal and lower gastrointestinal**	**27**	
○ Ileocecal resection	6	Senhance [5]; Hugo RAS [1]
○ Appendectomy	6	Senhance
○ Ladd’s procedure and appendectomy	4	Senhance
○ Caecostomy	3	Senhance
○ Anorectoplasty	2	Senhance
○ Exploratory laparoscopy for intestinal duplication	1	Senhance
○ Rectal pull-through coloanal anastomosis	1	Senhance
○ Proctocolectomy with IPAA	1	Senhance
○ Ileostomy	1	Senhance
○ Subtotal colectomy	1	Senhance
○ Pull-through procedure for Hirschsprung disease	1	Senhance
**Urologic, endocrine and pelvic tumor**	**14**	
○ Pyeloplasty	7	Senhance
○ Teratoma extirpation	2	Senhance
○ Abdominal orchidopexy	1	Senhance
○ Adrenalectomy	1	Hinotori
○ Nephrectomy with urethrectomy	1	Senhance
○ Urachal resection	1	Senhance
○ Paraganglioma resection	1	Senhance
**Thoracic**	**5**	
○ Lung sequestration resection	5	Senhance
**Total**	**166**	

The studies were conducted in Germany [16], the United States [17], Italy [18], Japan [19, 20], and in a multicenter cohort from the Netherlands and Germany [21]. One included article was an adult case series from which a single 17-year-old patient was extracted because he met the pediatric age criterion of this review [18].

### Robotic platforms and technical profile

The included studies reported three new-generation multiport robotic platforms: Senhance, Hugo RAS, and Hinotori. Platform distribution was markedly concentrated around Senhance, which was reported in 4 of 6 studies and accounted for 164 of 166 patients (98.8%) [16, 17, 19, 21]. Hugo RAS [18] and Hinotori [20] were each represented by 1 patient (0.6%), while no eligible pediatric clinical study with extractable patient-level data was identified for Versius, Revo-i, KangDuo, Toumai, Avatera, or Dexter.

**Fig. 2:**
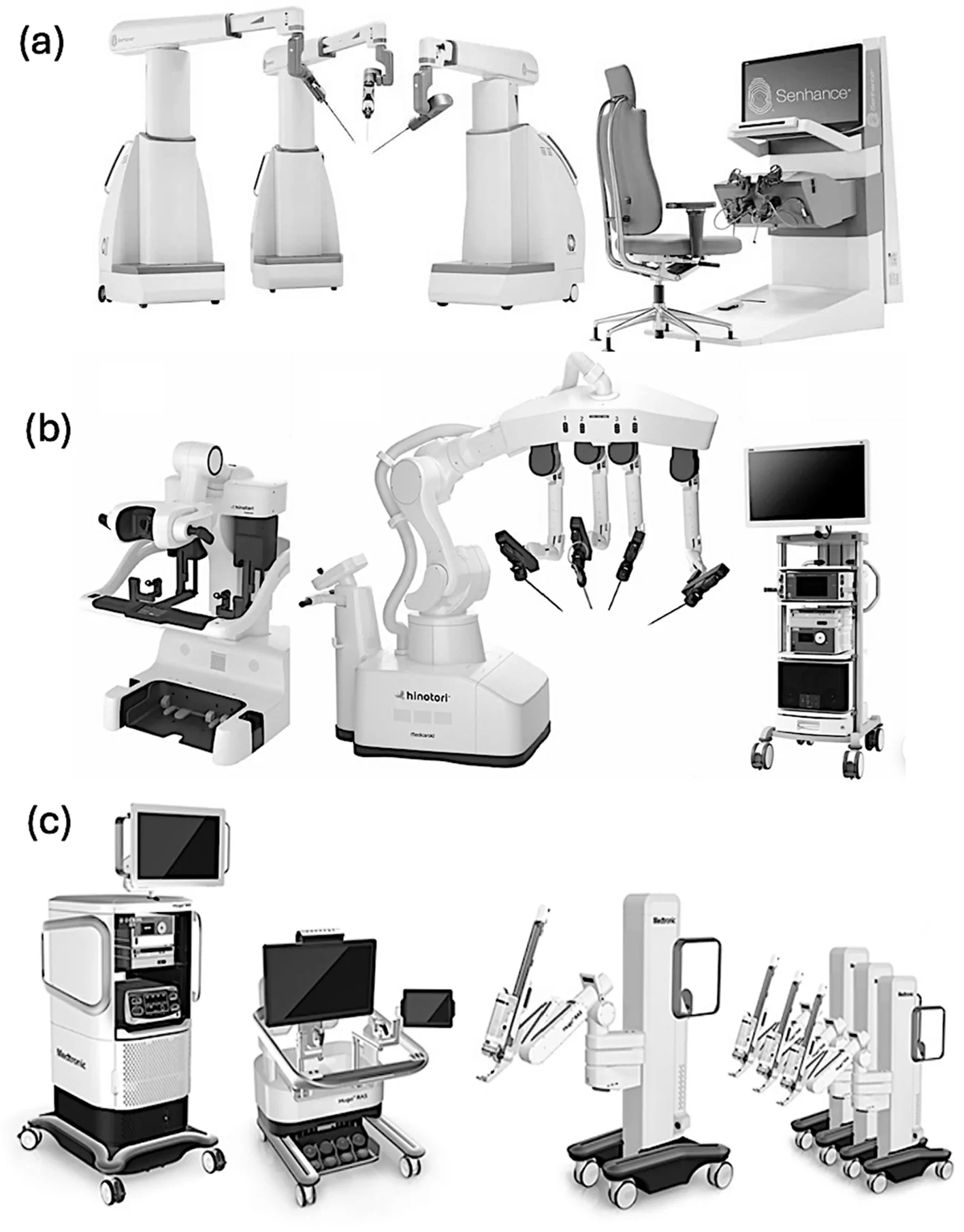
New-generation multiport robotic platforms reported in pediatric surgical practice. Representative systems included in the review: Senhance Surgical System **(a)**, Hinotori Surgical Robot System **(b)**, and Hugo RAS system **(c)**

The Senhance Surgical System (SSS; formerly Telelap ALF-X, then TransEnterix) is a modular multiport platform with CE marking and FDA clearance. Its architecture is based on three or four independent robotic arm carts, each mounted on a separate mobile unit and repositionable without undocking the whole system. This configuration allows flexible arm placement around the patient and facilitates access to the operative field or anesthetic area when required. The surgeon operates from an open console, maintaining visual contact with the operative team, while camera control is supported by an infrared eye-tracking system. In the included pediatric literature, Senhance represented the largest body of evidence and was the only platform used with robotic instruments smaller than 8 mm, including 3 mm reusable instruments and 5 mm instruments. The system also integrates with standard laparoscopic cameras, conventional trocars, and non-proprietary energy devices. Another distinctive feature is haptic feedback, provided through sensor-equipped handles with visual and acoustic alerts when force or motion thresholds are approached [16, 17, 19, 21] (Fig. 2a).

The Hinotori Surgical Robot System (Medicaroid Corporation, Kobe, Japan) is a Japanese-developed multiport robotic platform with domestic regulatory approval. It was designed with compact articulated robotic arms and an open-console configuration to support integration into conventional operating room workflows. The platform uses 8 mm robotic instruments and provides articulated motion intended to facilitate dissection and suturing in confined spaces. Preclinical studies have evaluated its ability to perform accurate suturing tasks in small operative cavities (Fig. 2b). In the included pediatric literature, Hinotori was represented by one child who underwent transperitoneal adrenalectomy. The reported case used three 8 mm robotic ports, together with assistant and retractor ports, and incorporated patient-specific preoperative 3D CT-based simulation to plan port placement and anticipate robotic arm movement [20].

The Hugo RAS system (Medtronic, Minneapolis, MN, USA) is a modular multiport platform that received CE marking for general surgery in October 2022. Its architecture consists of four independent arm carts, each positioned separately according to the surgical target and planned port layout. Before surgery, each arm requires manual adjustment of tilt and docking angles, which define the vertical inclination and horizontal working direction of the robotic arm. Hugo RAS uses 8 mm robotic instruments and a cart-based modular design, allowing different docking arrangements according to the operative quadrant. The surgeon operates from an open console using 3D polarized glasses and views the operative field on a shared high-definition screen, supporting direct communication with the operating room team and simultaneous visualization by other team members (Fig. 2c). In the included pediatric literature, Hugo RAS was represented by one adolescent patient who underwent ileocecal resection [18].

### Patient characteristics

The combined cohort of 166 patients was 56.6% male (n = 94) and 43.4% female (n = 72). Patient age at surgery ranged from 15 days to 17 years and body weight spanned 3.8 kg to over 100 kg across studies. The youngest patient in the cohort was a 15-day-old neonate undergoing resection of an intra-abdominal and intrapelvic sacrococcygeal teratoma (Altman type 4) and twenty-three patients in that cohort (15.1%) were younger than one year or weighed less than 10 kg and constituted a pre-specified small-child subgroup [21].

The Kato 2024 series operated exclusively on infants (mean age 7 months, range 5 – 9 months; mean weight 8 kg, range 7 – 9 kg), extending SSS use into the first year of life for pelvic reconstructive surgery [19].

### Procedural distribution

Across the 166 patients, 27 procedure categories were reported, and grouped into five surgical domains to improve interpretability while retaining procedure-level counts in Table 1: abdominal wall surgery (n = 48), upper gastrointestinal/hepatobiliary/splenic surgery (n = 72), colorectal/lower gastrointestinal surgery (n = 27), urologic/endocrine/pelvic tumor surgery (n = 14), and thoracic surgery (n = 5). The most frequent procedures were inguinal hernia repair in 48 (28.9%) patients, Nissen fundoplication in 40 (24.1%), cholecystectomy in 20 (12.0%), pyeloplasty in 7 (4.2%), ileocecal resection in 6 (3.6%), appendectomy in 6 (3.6%), lung sequestration resection in 5 (3%), and splenectomy or splenic cyst resection in 5 (3%). Ladd’s procedure with appendectomy was reported in 4 patients (2.4%), caecostomy in 3 (1.8%), Heller-Dor procedure in 3 (1.8%), anorectoplasty in 2 (1.2%), resection of esophageal duplication cyst in 2 (1.2%), and teratoma extirpation in 2 (1.2%). All 13 remaining procedures were reported in single patients (0.6%) (Table 1).

### Technical configuration

Technical reporting varied across studies. Senhance was used with 5 and 10 mm cameras and with 3 or 5 mm robotic ports [16, 17, 19, 21]. Kato et al. used a 10-mm 3D 4 K camera through a 12-mm camera channel, together with 3- and 5-mm instruments [19]. Hugo RAS [18] and Hinotori [20] used 8 mm cameras and 8 mm robotic ports. Senhance was the only platform in the included pediatric dataset used with robotic access smaller than 8 mm, while assistant access was reported in most studies (Table 2).

**Table 2:** Technical configuration and perioperative outcomes of included studies

Study	Platform	N	Main procedure[s]	Camera / robotic ports	Assistant access	Docking time	Operative time	Blood loss / transfusion	Length of stay	Conversion	Complications
Holzer et al., 2022 [16]	Senhance	1	Anderson-Hynes pyeloplasty	5 mm camera and port	Auxiliary port reported	NR	270 min	0 mL / no transfusion	7 days	0	Postoperative urinary tract infection
Puentes et al., 2023 [17]	Senhance	8	Inguinal hernia repair, cholecystectomy, orchidopexy, exploratory laparoscopy	5 and 10 mm cameras; 3 and 5 mm robotic ports	Reported	8.6 min, range 7–21	105 min, range 53–162	0 mL / no transfusion	6 same-day discharges; 2 planned overnight	1/8	2 external robotic arm collisions; no postoperative complications
Gangemi et al., 2023 [18]	Hugo RAS	1	Ileocecal resection	8 mm camera and ports	12 mm assistant access	7 min	205 min; console time 160 min	30 mL / no transfusion	7 days	0	0
Kato et al., 2024 [19]	Senhance	3	Anorectoplasty; rectal pull-through coloanal anastomosis	10-mm 3D 4 K camera through a 12-mm camera channel; 3- and 5-mm robotic instruments	Multichannel / assistant access reported	NR	428 min	0 mL / no transfusion	13 days	0	0
Okata et al., 2025 [20]	Hinotori	1	Transperitoneal adrenalectomy	8 mm camera and 3 ports	12 mm assistant port, 5 mm assistant port, 5 mm retractor port	NR	177 min; console time 84 min	0 mL / no transfusion	NR	0	0
Killaars et al., 2026 [21]	Senhance	152	Multiple abdominal, pelvic, urologic, colorectal, and thoracic procedures	5 mm camera, 3 and 5 mm robotic ports	Reported	9 ± 6 min	136 ± 87 min	NR / no transfusion reported	3 ± 5 days	18/152	4 procedure-related complications: 2 recognized intraoperatively and 2 diagnosed postoperatively, the latter included among 19 patients with 30-day postoperative complications; 7 reinterventions; 7 readmissions

Docking time was reported in three studies: 7 min in the Hugo RAS case, 8.6 min (range 7–21) in the Puentes et al. Senhance series, and 9 ± 6 min in the Killaars et al. Senhance cohort [17, 18, 21] (Table 2).

### Operative outcomes

Operative time was reported in all studies and varied widely across procedures. Mean operative time ranged from 105 min in the Puentes et al. series [17] to 428 min in the Kato et al. series [19], while the largest Senhance cohort reported 136 ± 87 min, combining procedures of different complexity (Table 2).

Estimated blood loss was negligible in all studies reporting this outcome and no transfusions were reported (Table 2).

Postoperative stay was reported heterogeneously. In Puentes et al., six of eight patients were discharged on the day of surgery and two had planned overnight admissions [17]. Holzer et al. and Gangemi et al. each reported a 7-day stay [16, 18], Kato et al. reported discharge on postoperative days 11–15 [19], and Killaars et al. reported a mean of 3 ± 5 days [21]. Length of stay was not reported for the Hinotori case [20] (Table 2).

### Conversions

Conversion was reported in 19 of 166 patients (11.4%; 95% CI, 7.5–17.2%). Eighteen events were reported in the Killaars et al. cohort (11.8%; 95% CI, 7.6–17.9%), including 17 conversions to conventional minimally invasive surgery (11.2%; 95% CI, 7.1–17.2%) and one conversion to open surgery (0.7%; 95% CI, 0.1–3.6%) [21]. Puentes et al. reported one anticipated escalation from robotic exploration to laparoscopy (12.5%; 95% CI, 2.2–47.1%) and then laparotomy because manual palpation was required to locate the lesion [17]. All 19 events arose in Senhance reports, which is expected given that Senhance accounted for 164 of 166 patients; this distribution does not support a platform comparison. In the prespecified small-child subgroup of Killaars et al. (< 1 year of age and/or <10 kg; n = 23), two patients (8.7%; 95% CI, 2.4–26.8%) were converted to conventional laparoscopy, one because of limited instrument mobility and one because additional visualization was required; none underwent open conversion [21]. Reasons for conversion were limited instrument motion or robotic arm collision in 10 cases, defective instrument in 3, poor visualization or concern for iatrogenic injury in 3, need for a CoolSeal device during thoracoscopic lung sequestration resection in 1, and adhesions in 1 (Table 2).

### Complications, reoperations, and readmissions

No deaths were reported in any included study (0/166, 0.0%; 95% CI, 0.0–2.3%). Four intraoperative complications were reported among 166 patients (2.4%; 95% CI, 0.9–6.0%). Puentes et al. [17] reported two external robotic arm collisions. Killaars et al. [21] reported two complications recognized intraoperatively: a suspected iatrogenic esophageal wall injury during Nissen fundoplication, which was preventively sutured, and bleeding during splenectomy requiring conversion to laparoscopy.

Across all included reports, 20 of 166 patients experienced a postoperative complication (12.0%; 95% CI, 7.9–17.9%). This total comprised 19 of 152 patients in the Killaars et al. cohort (12.5%; 95% CI, 8.2–18.7%) and one patient with a urinary tract infection after Senhance pyeloplasty [16]. In the Killaars et al. cohort, two procedure-related complications were diagnosed postoperatively: a thermal gastric perforation requiring laparoscopic re-exploration on postoperative day 3 (Clavien grade 4a) and a cystic duct leak diagnosed following postoperative readmission and treated endoscopically. Both events were included among the 19 postoperative complications reported in that cohort. Seven patients required reoperation or reintervention (4.6%; 95% CI, 2.2–9.2%), and seven were readmitted within 30 days (4.6%; 95% CI, 2.2–9.2%) (Table 2). Furthermore, for analyses of conversion and postoperative complications, period 1 comprised cases 1–56 at the first center and cases 1–20 at the second center (n = 76), whereas period 2 comprised cases 57–112 at the first center and cases 21–40 at the second center (n = 76). Postoperative complications occurred in 14 patients in period 1 (18.4%, 95% CI, 11.3–28.6%) and five patients in period 2 (6.6%, 95% CI, 2.8–14.5%), deriving from a possible learning-curve effect on overall system management, however because no formal between-period test was reported for this outcome, this observation should be considered hypothesis-generating.

### Training, proctoring, and implementation

Training or implementation preparation was described in the studies, but reporting was heterogeneous. Holzer et al. specified two days of dry-laboratory training, one day of wet-laboratory training, and a written certification test [16]. Puentes et al. reported online pre-learning modules, a one-day dry-laboratory course, a one-day wet-laboratory course, and three proctored clinical cases before independent use [17]. Gangemi et al. reported platform certification at a Medtronic-designated training facility, without stating the duration or a proficiency threshold [18]. Kato et al. described platform training and preoperative rehearsal of arm, instrument, and patient positioning, but did not report a case-based competency threshold [19]. Killaars et al. reported prior minimally invasive and robotic experience among the operating surgeons and senior-surgeon staffing, without quantifying prior case volume [21]. Okata et al. used patient-specific 3D CT simulation, infant-sized preclinical models, and multidisciplinary planning, but did not report training duration or the number of cases required for proficiency [20]. No included study prospectively defined a competency endpoint or estimated a transferable case number for proficiency.

## Discussion

This systematic review summarizes the currently published pediatric experience with new-generation multiport robotic platforms beyond the da Vinci system [8, 9]. Although several platforms have entered adult surgical practice, pediatric evidence remains limited. Only three systems were represented in the included studies: Senhance, Hugo RAS, and Hinotori. Among 166 pediatric patients, Senhance accounted for 164 cases [16, 17, 19, 21], whereas Hugo RAS [18] and Hinotori [20] were each represented by a single case. No eligible pediatric clinical data were identified for Versius, Revo-i, KangDuo, Toumai, Avatera, or Dexter. The current literature therefore does not provide a balanced comparison among platforms but describes an early and highly asymmetric phase of pediatric adoption. To our knowledge, this is the first systematic review to examine the pediatric experience with new-generation multiport robotic platforms other than the da Vinci system.

The predominance of Senhance is a central finding of this review, and it is not only numerical but also technical. Senhance was the only platform in the included dataset used with robotic access smaller than 8 mm, including 3 mm and 5 mm instruments compatible with standard laparoscopic trocars [16, 17, 19, 21]. This is particularly relevant in pediatric surgery, where body size, working space, abdominal wall surface, and inter-port distance influence feasibility [1, 2, 6]. Use in infants and children weighing less than 10 kg was reported only with Senhance [17, 19, 21]. By contrast, Hugo RAS and Hinotori were reported in larger pediatric patients and used 8 mm robotic ports [18, 20]. These data indicate that Senhance currently has the widest documented pediatric size range among the included systems. The evidence for Hugo RAS and Hinotori remains preliminary. Hugo RAS was reported in a 17-year-old patient undergoing ileocecal resection [18], while Hinotori was reported in an 11-year-old child undergoing adrenalectomy [20]. Both cases show successful use in selected pediatric-age patients, but they do not allow conclusions on broader feasibility, safety, or suitability in younger children. Their current pediatric applicability appears limited by the use of 8 mm robotic instruments, even if the da Vinci Surgical System was similarly constrained in its early pediatric adoption phase by the use of 8 mm instruments, with small body cavity dimensions identified as the primary limiting factor for robotic triangulation and patient selection criteria initially excluding children below 10–15 kg, a barrier that was progressively overcome with the growing experience and surgical volume [22–24].

The procedural spectrum was broad, with 27 procedure categories across abdominal, pelvic, urologic, colorectal, and thoracic surgery, showing early exploration across different pediatric indications. However, most procedures were represented by small numbers or isolated cases, and the largest cohort included multiple procedure types [21]. This distribution matches the same trajectory documented for the da Vinci during its first decade of paediatric adoption, in which over 2,300 procedures spanning multiple surgical subspecialties were accumulated before comparative effectiveness could be established for a limited number of specific indications [25], therefore, the current evidence is better interpreted as a map of early applications.

Technical reporting, including camera and instrument size, robotic arm configuration, assistant access, docking time, and console time were not uniformly reported. In pediatric robotic surgery, these variables are essential because they determine whether a platform can be adapted to small operative fields and reproduced by other centers. The IDEAL framework for surgical robotics specifically identifies standardized technical reporting at early development stages as a prerequisite for reproducibility, cross-platform comparison, and progressive safety evaluation, therefore future pediatric studies should include standardized technical reporting [26].

Conversions occurred in 19 of 166 patients and were reported only in Senhance studies [17, 21]. This finding should be interpreted in relation to the distribution of the evidence, since Senhance accounted for nearly all included patients. Reported reasons included limited instrument motion, external arm collision, defective instruments, poor visualization, concern for iatrogenic injury, adhesions, need for non-robotic energy devices, and need for manual palpation. These causes highlight the importance of pediatric-specific port planning, adequate arm spacing, suitable instruments, and clear thresholds for conversion. These causes are consistent with those previously documented in early paediatric robotic cohorts using the da Vinci platform, where technical instrument limitations and inter-arm collisions represent the most frequently identified precipitating factors for conversion [27, 28].

No mortality was reported, and intraoperative complications were few [16, 17, 21]. Most postoperative complications and reinterventions came from the largest multicenter Senhance cohort [21]. However, the absence of comparative pediatric studies prevents conclusions about superiority, equivalence, or non-inferiority compared with conventional laparoscopy, open surgery, or established robotic systems [29–31].

Training and implementation were recurring elements. Several studies described structured training, previous robotic experience, proctoring, technical support, or preoperative simulation [16–21]. These aspects are particularly relevant in children, where reduced working space and narrow margins for technical error increase the need for careful preparation [6, 7]. The Hinotori case illustrates the value of patient-specific preoperative simulation [20], while the Senhance experience suggests that docking and setup improve with institutional familiarity [21].

Current evidence supports only cautious, center-specific introduction of these platforms. Centers considering adoption should match instrument and camera diameter to patient size, plan port and cart geometry for each procedure, maintain unobstructed access to the patient, use structured team training and proctoring, and define conversion thresholds before clinical use. Senhance has the broadest published pediatric experience and the only reported sub-8-mm robotic access, but these observations do not demonstrate superiority. Evidence is insufficient to recommend one new-generation platform over another or over conventional laparoscopy, open surgery, or da Vinci systems. Initial use should occur within prospective registries or standardized institutional protocols that capture procedure-specific technical and clinical outcomes.

This review has several limitations. The evidence base was small, recent, and mainly descriptive. Most studies were case reports or small case series, creating substantial susceptibility to publication bias and selective outcome reporting, while comparator groups were absent. One multicenter cohort contributed 152 of 166 patients overall and 152 of 164 Senhance cases, so aggregate estimates largely reflect that study. Procedures, patient sizes, technical configurations, and outcome definitions were heterogeneous, which limits the interpretability of aggregate descriptive statistics. Restriction to English-language publications introduces potential language bias and may have excluded reports from regions where Hinotori, Revo-i, and other platforms were developed or first adopted. Follow-up was limited, and long-term functional outcomes were incompletely reported. Differential regional adoption, market penetration, and publication practices may also have influenced the observed platform distribution.

## Conclusion

Current pediatric evidence on new-generation multiport robotic platforms is early, descriptive, and highly concentrated. Senhance accounts for most published cases and all reported sub-8-mm robotic access, but one multicenter cohort dominates these observations, and differential adoption, market penetration, and publication practices may contribute to the distribution. Hugo RAS and Hinotori are represented by one pediatric case each. The available evidence does not support comparative claims regarding safety, effectiveness, suitability, or superiority. Prospective multicenter studies should use procedure-specific comparators and standardized reporting of patient size, port geometry, arm configuration, training, conversions, and complications.

## Supplementary Information

Below is the link to the electronic supplementary material.

Supplementary file1 (DOCX 270 KB)

Supplementary file2 (DOCX 19 KB)

## Data Availability

No datasets were generated or analysed during the current study.
