# Supplementary material for: Beyond da Vinci: a systematic review of next-generation multiport robotic platforms in pediatric surgery
Source: J Robot Surg. 2026 Sep 7;20(1):968. doi: 10.1007/s11701-026-03879-4 (PMC13550092; doi:10.1007/s11701-026-03879-4)
Supplement: Supplementary file 2 [file 11701_2026_3879_MOESM2_ESM.docx]

Supplementary Material

Table 1. Risk of bias assessment of the six included studies.

|  | **Holzer 2022** | **Puentes 2023** | **Gangemi 2023** | **Kato 2024** | **Okata 2025** | **Killaars 2026** |
| --- | --- | --- | --- | --- | --- | --- |
| **JBI Critical Appraisal Checklist for Case Reports (8 items)** | | | | | | |
| 1. Demographic characteristics clearly described? | Y | — | — | — | Y | — |
| 2. Patient history and timeline clearly described? | Y | — | — | — | Y | — |
| 3. Clinical condition on presentation clearly described? | Y | — | — | — | Y | — |
| 4. Diagnostic tests/assessment methods clearly described? | Y | — | — | — | Y | — |
| 5. Intervention/procedure clearly described? | Y | — | — | — | Y | — |
| 6. Post-intervention clinical condition clearly described? | Y | — | — | — | U | — |
| 7. Adverse events identified and described? | Y | — | — | — | Y | — |
| 8. Case report provides takeaway lessons? | Y | — | — | — | Y | — |
| **Overall judgment** | **Low** | — | — | — | **Low** | — |
| **JBI Critical Appraisal Checklist for Case Series (10 items)** | | | | | | |
| 1. Clear criteria for inclusion defined? | — | U | Y | U | — | — |
| 2. Condition measured in a standard, reliable way for all participants? | — | Y | Y | Y | — | — |
| 3. Valid methods used for identification of condition? | — | Y | Y | Y | — | — |
| 4. Consecutive inclusion of participants? | — | U | Y | U | — | — |
| 5. Complete inclusion of participants? | — | U | Y | U | — | — |
| 6. Clear reporting of demographics of participants? | — | Y | Y | Y | — | — |
| 7. Clear reporting of clinical information? | — | Y | U | Y | — | — |
| 8. Outcomes/follow-up results clearly reported? | — | Y | Y | Y | — | — |
| 9. Clear reporting of presenting site/clinic information? | — | Y | Y | Y | — | — |
| 10. Statistical analysis appropriate? | — | NA | NA | NA | — | — |
| **Overall judgment** | — | **Moderate** | **Low** | **Moderate** | — | — |
| **ROBINS-I (7 domains)** | | | | | | |
| D1. Bias due to confounding | — | — | — | — | — | **Moderate** |
| D2. Bias in selection of participants | — | — | — | — | — | **Moderate** |
| D3. Bias in classification of interventions | — | — | — | — | — | **Low** |
| D4. Bias due to deviations from intended interventions | — | — | — | — | — | **Low** |
| D5. Bias due to missing data | — | — | — | — | — | **Moderate** |
| D6. Bias in measurement of outcomes | — | — | — | — | — | **Moderate** |
| D7. Bias in selection of the reported result | — | — | — | — | — | **Low** |
| **Overall judgment** | — | — | — | — | — | **Moderate** |

*Y = Yes; N = No; U = Unclear; NA = Not Applicable.*

*Low = low risk; Moderate = moderate risk; Serious = serious risk.*
